# Designing robot-to-robot interaction to support early-stage engagement in a teleoperated Robot Avatar system with multiple robots

**DOI:** 10.3389/frai.2026.1928110

**Published:** 2026-10-12

**Authors:** Kiichiro Suemitsu, Midori Ban, Hideyuki Takahashi, Hiroshi Ishiguro, Takamasa Iio, Keito Izawa, Yurina Someya, Sonia Yuhui Zhang, Keiko Yukita, Shogo Nishimura, Yukiko Horikawa, Takahiro Miyashita

**Affiliations:** 1 Graduate School of Engineering Science, The University of Osaka, Toyonaka, Japan; 2 Department of Robotics, Faculty of Engineering, Kyoto Tachibana University, Kyoto, Japan; 3 Faculty of Science and Engineering, Otemon Gakuin University, Ibaraki, Japan; 4 Faculty of Culture and Information Science, Doshisha University, Kyotanabe, Japan; 5 Graduate School of Informatics, Nagoya University, Nagoya, Japan; 6 Department of Anthropology, The New School for Social Research, New York, NY, United States; 7 i-RooBO Network Forum, Osaka, Japan; 8 Deep Interaction Laboratory Group, Advanced Telecommunications Research Institute International (ATR), Kyoto, Japan

**Keywords:** inter-robot interaction, persuasive interaction, social presence, teleoperated Robot Avatar, user engagement

## Abstract

Teleoperated Robot Avatars enable operators to provide customer service from remote locations. Effective persuasive interaction requires more than information presentation; customers' attention and engagement must be elicited before their attitudes and behavior can be influenced. Social presence may support this type of engagement. Based on prior research, we conceptualize a Robot Avatar's social presence as the extent to which customers perceive it, i.e., not as a machine or information medium, but as a social entity customers can attend to and interact with. Therefore, we examined a design approach intended to enhance a Robot Avatar's social presence by forming a social context around it. We developed GayaRobo, comprising one teleoperated Robot Avatar and two supporting Gaya robots. Rather than approaching users as independent agents, the Gaya robots respond to the Robot Avatar's utterances via speech and movement, thereby forming a social context centered on the avatar. We conducted a field experiment at a commercial complex in Osaka, Japan, to compare observable early-stage engagement behaviors of passersby toward the Robot Avatar across three experimental conditions. The same three-robot configuration was used under three conditions: the Gaya robots responded to the Robot Avatar, only the Robot Avatar spoke, or only the Gaya robots spoke. Two annotators coded four behaviors among 1,379 passersby in 270 min of video recordings: looking at the Robot Avatar, approaching it, talking to it, and picking up a flyer. After correction for multiple testing, only approaching the Robot Avatar was significantly associated with experimental condition and occurred more frequently than expected under the inter-robot interaction condition. These findings provide behavioral evidence that the inter-robot interaction condition was associated with approaching the Robot Avatar, an observable indicator of early-stage engagement. Because social presence was not directly measured, the findings did not demonstrate that GayaRobo enhanced the Robot Avatar's social presence. Nevertheless, they highlight approach behavior as a relevant observable outcome for future evaluations of designs that consider the social context surrounding a Robot Avatar in customer service settings.

## 1 Introduction

Teleoperated Robot Avatars have attracted attention as a technology that enables operators to provide interpersonal services to customers from remote locations by using robots installed at service sites. To date, their use has been investigated in various customer service settings, including customer service in cafés (Takeuchi et al., 2020), guidance and information provision in commercial facilities (Kanda et al., 2010; Baba et al., 2021), and product sales support (Song et al., 2021). Remote service provision offers the advantage of operators participating in work from a specific location. Accordingly, it can expand employment opportunities for people with physical limitations (Takeuchi et al., 2020; Kanetsuna et al., 2022) and enable diverse human resources to work beyond geographical and physical constraints (Ferreira et al., 2021; Frankiewicz and Chamorro-Premuzic, 2020). Conversely, to ensure that teleoperated Robot Avatars function effectively in customer service settings, it is important to design persuasive interactions that present information, attract customers' attention, engage them, and influence their attitudes and behaviors toward the service (Skalski and Tamborini, 2007; Van Doorn et al., 2017).

In these types of persuasive interactions, eliciting users' attention and engagement serves as an important pre-requisite for subsequent attitude formation and behavioral change. Indeed, past research on service robots has shown that customer engagement generated via interactions with robots is related to customers' intention to continue using the service (Hyun et al., 2022). One factor related to this engagement is social presence. Persuasive research using interactive agents has shown that social presence enhances engagement, such as users' message processing, and is related to subsequent attitudes and behavioral intentions (Skalski and Tamborini, 2007). In addition, for technologies introduced into service settings, such as stores and commercial facilities, the concept of automated social presence has been proposed, suggesting that technologies that evoke social presence are related to customers' service experiences (Van Doorn et al., 2017). Recent service-robot research further suggests that automated social presence is related to consumers' perceived service value and intention to use service robots (Flavián et al., 2024). Therefore, based on the concept of social presence discussed in past literature (Biocca et al., 2003; Oh et al., 2018), the social presence of teleoperated Robot Avatars is conceptualized in this study as the extent to which customers perceive a Robot Avatar not merely as a machine or an information presentation medium, but also as a social entity to which they can direct attention and with which they can engage in interaction. Taken together, examining design methods for enhancing the social presence of teleoperated Robot Avatars is an important research topic for realizing persuasive interactions that promote users' attention and engagement in customer service settings.

In this study, we focus on interaction among multiple robots as a design approach intended to enhance the social presence of teleoperated Robot Avatars. In conventional teleoperation, a one-to-one operation method, in which one operator controls one robot, has typically been used. In contrast, previous studies have shown that using multiple robots or agents can generate greater social influence than using a single robot or agent. For example, the effects of being praised by multiple agents (Shiomi et al., 2020) and the persuasiveness of suggestions made by multiple robots in public spaces (Tae et al., 2021) have been examined in previous studies. The use of multiple robots has also been investigated in recommendation settings involving teleoperated Robot Avatars. For instance, Hatano et al. (2024) examined the effects of expressions by multiple Robot Avatars on robot influence in a real-world recommendation setting, showing that multiple robots collaboratively approaching users can enhance the influence of the overall system and promote purchasing behavior. These studies suggest that using multiple robots or agents has the potential to enhance user engagement and the social influence of robots or agents.

However, most of the previously conducted research using multiple robots has examined how multiple robots, each acting as an independent agent, approach users and influence their responses and behaviors. In contrast, when teleoperated Robot Avatars are used in real-world customer service settings, it is important that they are perceived not as information media but as social interaction partners to which users direct their attention and with which they can engage in interactions. To this end, it is necessary to design teleoperated Robot Avatars in ways that enhance their social presence, which supports users' engagement. However, design methods for enhancing the social presence of teleoperated Robot Avatars involving the formation of a social context around them have not yet been sufficiently investigated.

Therefore, this study proposes a design approach intended to enhance the social presence of a teleoperated Robot Avatar by forming a social context through robot-to-robot interaction centered on the Robot Avatar, rather than having multiple robots independently approach users. To examine this design approach, the present study focuses on observable early-stage engagement behaviors of passersby toward the Robot Avatar rather than directly assessing their perceptions of the Robot Avatar's social presence. Specifically, robots that respond to the operator's utterances were placed around the Robot Avatar operated by the operator. In this study, we referred to these surrounding robots as Gaya robots. Gaya robots do not approach users as independent persuasive agents. Instead, by responding to the Robot Avatar's utterances via speech and movement, they play a role in forming a social context centered on the Robot Avatar. Prior work on multiple social robots suggests that robot-to-robot interaction can be used to present roles and social relationships among robots to observers (Shiomi, 2024). This perspective is relevant to public service settings, where users' experiences of service robots can be shaped by nearby others and by the public visibility of the interaction (Tretter et al., 2025). Thus, in this study, robot-to-robot interaction is treated not merely as coordination among robots, but as a way to form a visible social context around the Robot Avatar. Within this social context, the Gaya robots' responses were intended to present the Robot Avatar as the target of the surrounding robots' attention and responses. This design rationale draws on the concept of social proof, which suggests that people are more likely to direct their attention toward objects that others are attending to (Cialdini, 2013). A similar attentional pattern has also been reported for objects attended to by robots; for example, robots looking at digital advertisements have been shown to increase passersby's attention to those advertisements (Amada et al., 2023). Accordingly, social proof served as one theoretical rationale for the design. However, the present study did not assess whether passersby perceived the interaction in this way.

In this study, we referred to the proposed system consisting of a Robot Avatar and Gaya robots as GayaRobo. The term “Gaya” means liveliness or a bustling atmosphere in Japanese. To examine GayaRobo in terms of observable early-stage engagement behaviors, we compared three conditions in a real-world environment within a commercial facility. The first condition was the inter-robot interaction condition, in which the Robot Avatar spoke and the Gaya robots responded to its utterances. The second condition was the no inter-robot interaction condition, in which the Robot Avatar operated while the Gaya robots were present but did not respond to the Robot Avatar's utterances. The third condition was the no avatar-centered social context condition, in which the Gaya robots engaged passersby while the Robot Avatar was present but did not operate. By comparing the results obtained under these conditions, we examined whether the occurrence of observable early-stage engagement behaviors toward the Robot Avatar differed among the three experimental conditions. Specifically, we used behavioral indicators, such as looking at the Robot Avatar, approaching it, talking to it, and picking up a flyer, to evaluate behaviors involved in the early process of passersby's engagement with the Robot Avatar.

The contribution of this study lies in proposing the formation of a social context through robot-to-robot interaction centered on the Robot Avatar as a design approach intended to enhance the Robot Avatar's social presence, and in empirically examining the association between the inter-robot interaction condition and observable early-stage engagement behaviors of passersby in a real-world customer service setting. Furthermore, by examining the proposed approach in a real-world environment within a commercial facility through behavioral observations of passersby's early-stage engagement, this study provides theoretical and practical implications for persuasive interaction design concerned with early-stage user engagement, as well as for the design of next-generation persuasive interaction systems that consider social behavior in their operation.

## 2 Proposed system: GayaRobo

### 2.1 System overview

The proposed system, i.e., GayaRobo, consisted of one teleoperated Robot Avatar and two Gaya robots that responded to the Robot Avatar's utterances (Figures 1, 2). Rather than approaching passersby as independent agents, the Gaya robots served as supporting entities that responded to the Robot Avatar via speech and movement, thereby forming a social context centered on the Robot Avatar. Through this robot-to-robot interaction, the system was designed to present the Robot Avatar as the target of the surrounding robots' attention and responses within a visible social context.

**Figure 1:**
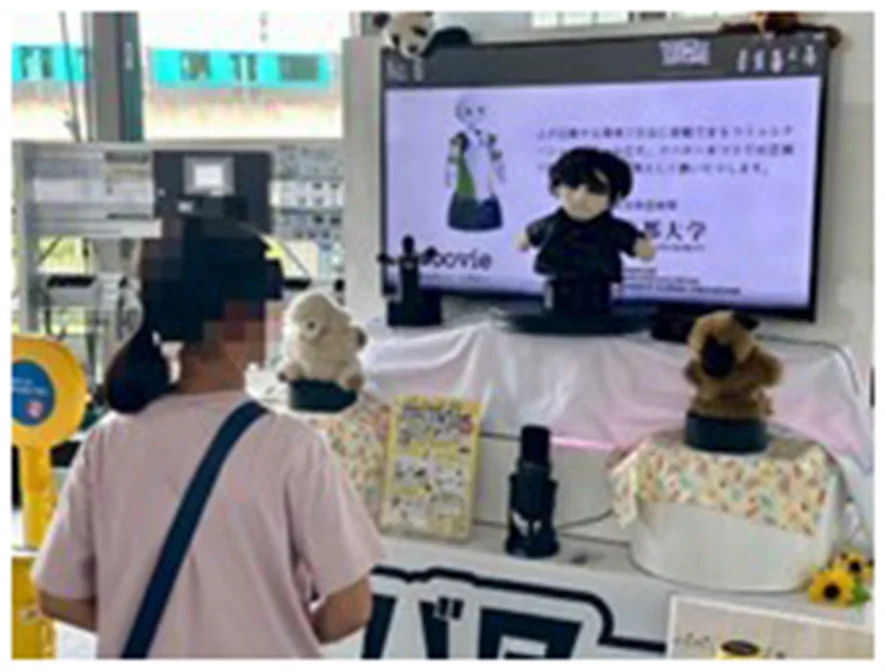
Display of the proposed GayaRobo system. The Robot Avatar was positioned at the center with one Gaya robot placed on each side. Reproduced from Figure 2 in Suemitsu et al. (2024), https://ipsj.ixsq.nii.ac.jp/records/231631, with permission from the Information Processing Society of Japan (IPSJ). Copyright © 2024 IPSJ.

**Figure 2:**
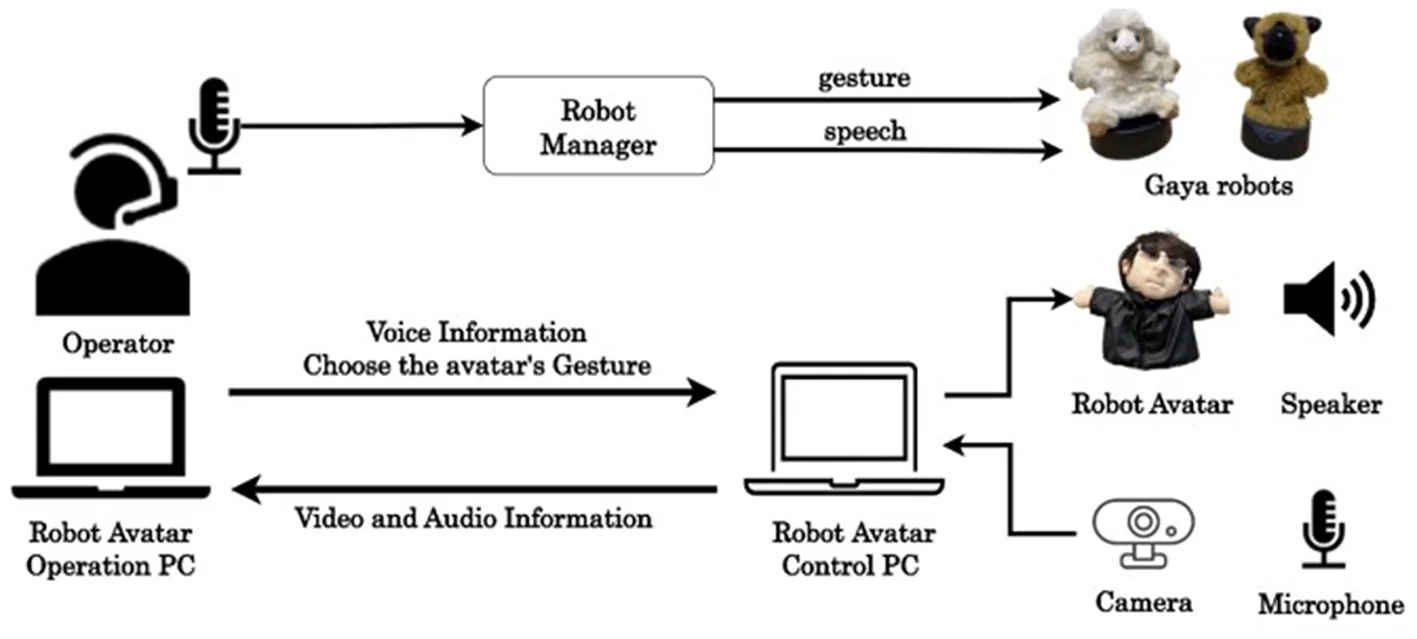
System architecture of GayaRobo.

For both the Robot Avatar and the Gaya robots, we used Kurumi-chan, a general-purpose communication robot developed by Vstone Co., Ltd. Kurumi-chan has a skeletal frame that can be fitted with various puppet-like coverings. The Robot Avatar was teleoperated using the Cybernetic Avatar Platform developed by the Advanced Telecommunications Research Institute International (ATR) (Horikawa et al., 2023).

During system operation, the Robot Avatar was positioned at the center, with one Gaya robot placed on each side (Figure 1). This arrangement was intended to make the Gaya robots' responses via speech and movement appear to be directed toward the Robot Avatar at the center. The three robots were also fitted with different puppet-like coverings. The Robot Avatar had the appearance of a man wearing black clothing and sunglasses, whereas the two Gaya robots had the appearances of a sheep and a dog. All three puppet-like coverings were selected from those available at the time of the experiment and were not chosen to elicit psychological or behavioral responses associated with their specific appearances.

### 2.2 Teleoperation of the Robot Avatar

As shown in Figure 2, the system was operated using a Robot Avatar's Operation personal computer (PC) located on the operator's side and a Robot Avatar's Control PC located on the Robot Avatar's side. The Robot Avatar's motion commands and the operator's audio were transmitted from the Robot Avatar's Operation PC to the Robot Avatar's Control PC. In the opposite direction, video and audio from the experimental site were transmitted from the Robot Avatar's Control PC to the Robot Avatar's Operation PC.

The Robot Avatar's movements were controlled using a browser-based interface on the Cybernetic Avatar Platform (Figure 3). When the operator selected a pre-registered gesture on the interface, the corresponding motion command was transmitted to the Robot Avatar via the Robot Avatar's Control PC. The system provided 13 movements, including raising the left hand, raising both hands, turning to the left, waving, and bowing.

**Figure 3:**
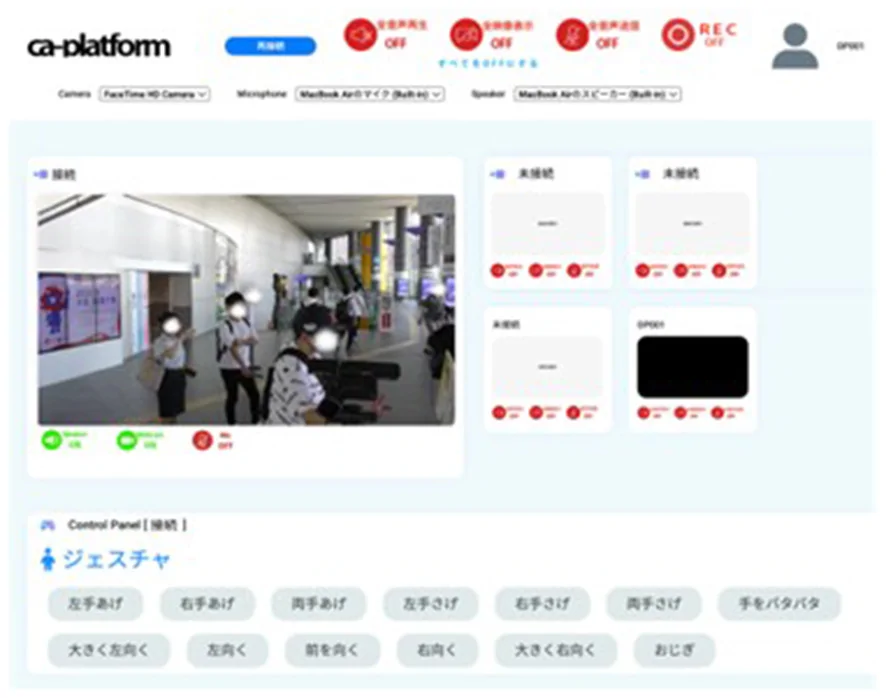
Browser-based interface used to operate the Robot Avatar.

The operator's speech was captured via a headset worn by the operator. The captured audio was transmitted in real time from the Robot Avatar's Operation PC to the Robot Avatar's Control PC and played through a speaker on the Robot Avatar's side. Hereafter, the operator's speech played from the Robot Avatar's side is referred to as the Robot Avatar's utterances.

A webcam and a condenser microphone were installed on the Robot Avatar's side to allow the operator to monitor the experimental site. Video from the webcam was displayed on the Cybernetic Avatar Platform interface running on the Robot Avatar's Operation PC. The operator used this video to operate the Robot Avatar while monitoring approaching passersby. The position of the webcam is shown in Figure 4. The condenser microphone, installed between the two Gaya robots, captured the voices of passersby. The captured audio was transmitted in real time from the Robot Avatar's Control PC to the Robot Avatar's Operation PC and played through the operator's headset. This configuration enabled bidirectional voice communication between the operator and passersby through the Robot Avatar.

**Figure 4:**
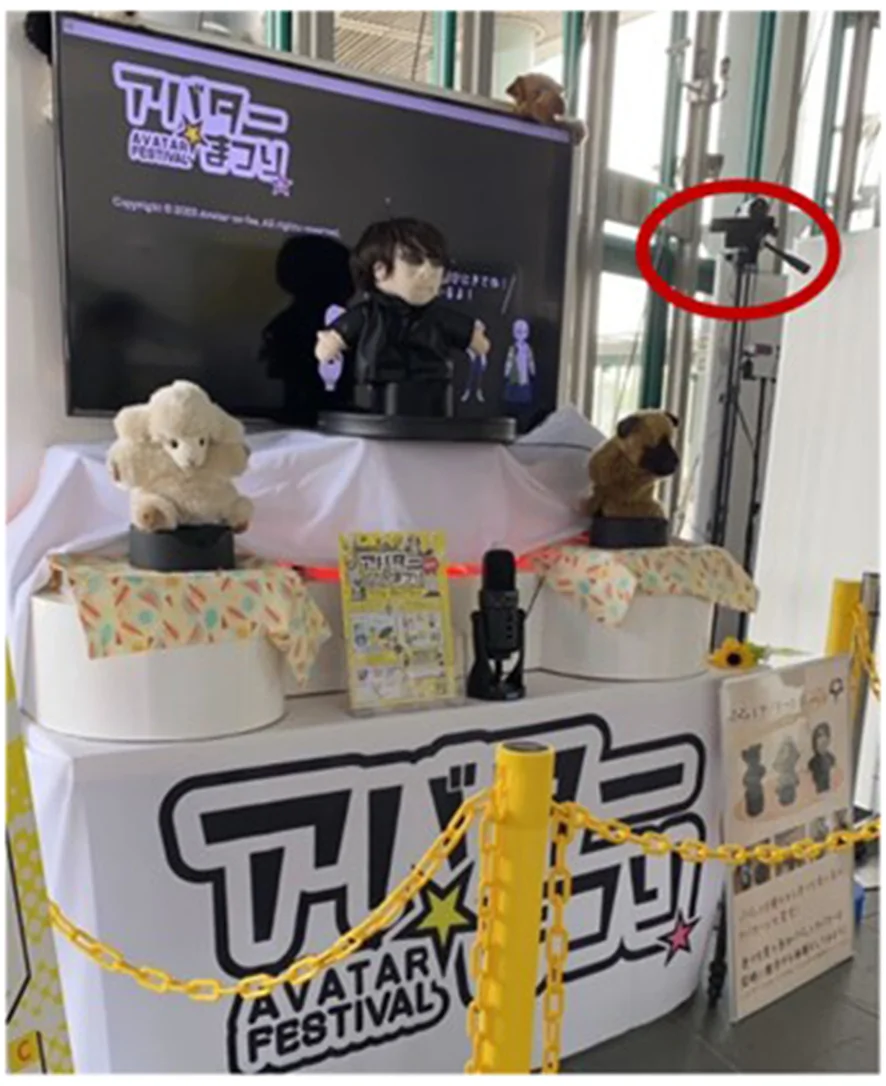
Position of the webcam.

### 2.3 Control of Gaya robot responses

The Gaya robots were designed to respond automatically through speech and movement after the end of a Robot Avatar utterance was detected, rather than speaking independently to passersby. Thus, the operator did not need to control the Gaya robots individually. The Gaya robots performed idle movements during normal operation and temporarily stopped these movements when they executed a response.

The audio signal captured via the operator's headset was used to detect the Robot Avatar's utterances. The input audio was acquired in monophonic mode at a sampling rate of 44.1 kHz with 16-bit quantization and processed in segments of 1,024 samples. For each segment, the system converted the audio data into 16-bit integer samples and calculated the mean absolute amplitude. This value was used as the amplitude measure for detecting the Robot Avatar's utterances. The onset of an utterance was detected when this amplitude measure exceeded a threshold. After at least 0.5 s had elapsed from the detected onset, the utterance was considered to have ended when the amplitude measure first fell to or below the threshold.

Before the experiment, we tested the system in the actual acoustic environment and tuned the threshold to 1,100 to reliably detect the operator's speech while suppressing false detections caused by background noise. This threshold was applied to the mean absolute amplitude calculated from audio samples acquired as 16-bit integers.

When the end of an utterance was detected, the idle movement was stopped. If at least 3.0 s had elapsed since the detected onset, the response-control program selected one of nine pre-recorded audio clips at random, played the clip, generated a beat movement matched to the clip duration, and executed the movement. The clips contained short expressions praising the Robot Avatar, such as “Amazing!” and “Genius!” After this process, the idle movement was resumed. The selection procedure did not prevent consecutive repetition; for example, the previously played clip was not excluded from the candidates.

## 3 Experiment

### 3.1 Field setting and experimental design

We conducted a field experiment at the Asia and Pacific Trade Center, a commercial complex in Osaka, Japan, as part of the “Avatar Festival” held in July 2023. The experiment compared the occurrence of observable early-stage engagement behaviors toward the Robot Avatar across the three experimental conditions in a real-world environment. The three system conditions were implemented during separate observation periods.

### 3.2 Experimental conditions

Figure 5 illustrates the three experimental conditions. The physical arrangement and the puppet-like coverings used for the three robots were identical across all three conditions. In every condition, the Robot Avatar remained at the center, with one Gaya robot positioned on each side. The conditions differed in terms of which robots produced utterances and whether the Gaya robots responded to the Robot Avatar.

**Figure 5:**
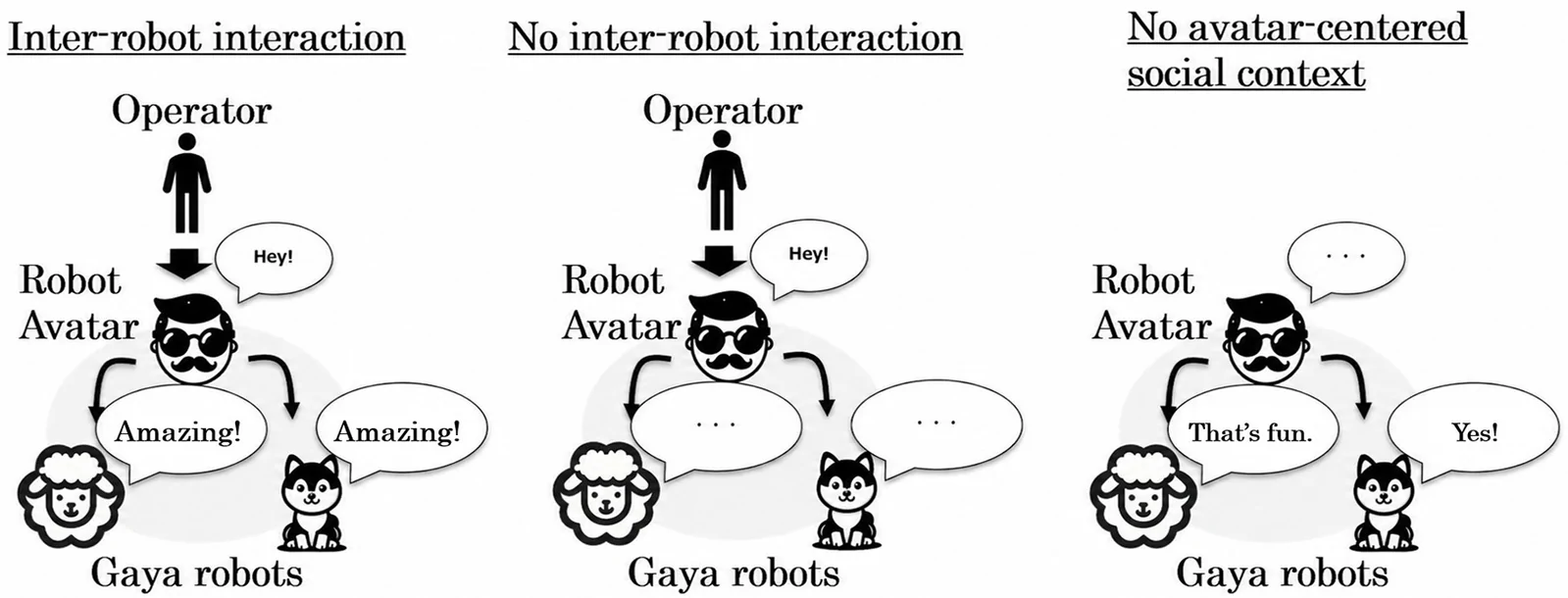
Conceptual representations of the three experimental conditions. Adapted from Figure 3 in Suemitsu et al. (2024), https://ipsj.ixsq.nii.ac.jp/records/231631, with permission from the Information Processing Society of Japan (IPSJ). Copyright © 2024 IPSJ.

Under the *inter-robot interaction condition*, the Robot Avatar was teleoperated to interact with passersby, and the Gaya robots responded to its utterances through speech and movement as described in Section 2.3. In the *no inter-robot interaction condition*, the Robot Avatar was similarly teleoperated and spoke to passersby, but the Gaya robots did not respond. In the *no avatar-centered social context condition*, the Robot Avatar was not operated and did not speak, whereas the Gaya robots produced pre-prepared utterances about topics related to the commercial complex. Throughout each 30-min observation period in this condition, the Gaya robots delivered a succession of pre-prepared utterances, with no intervals during which both robots were silent.

The latter two conditions were designed as comparison conditions under which no robot-to-robot interaction occurred; these conditions were therefore not intended to form a social context centered on the Robot Avatar. Instead, they represented two different forms of interaction without this type of social context; among the three robots, only the Robot Avatar spoke in the no inter-robot interaction condition, whereas only the Gaya robots spoke in the no avatar-centered social context condition.

### 3.3 Procedure

The experiment was conducted during the event on three weekdays: July 12, July 13, and July 21, 2023. On each day, data were collected in a corridor of the commercial complex from 13:00 to 14:30. Each daily session comprised three consecutive 30-min periods, with each of the three conditions implemented once. The order of the three conditions was rotated across the experimental days so that each condition was implemented once in each of the three time slots. The order was inter-robot interaction, no inter-robot interaction, and no avatar-centered social context on July 12; no inter-robot interaction, no avatar-centered social context, and inter-robot interaction on July 13; and no avatar-centered social context, inter-robot interaction, and no inter-robot interaction on July 21. Thus, 90 min of video data were collected under each condition, yielding a total observation time of 270 min.

The study was approved by the ethics review committee of the Advanced Telecommunications Research Institute International.

### 3.4 Behavioral measures and video annotation

Video recordings of the experimental site were used to determine whether passersby exhibited observable behaviors used as indicators of early-stage engagement with the Robot Avatar. To reduce the likelihood of counting the same person more than once, the analysis included only passersby who walked from the direction of the nearest station toward the GayaRobo exhibition area, as indicated by the arrows in Figure 6.

**Figure 6:**
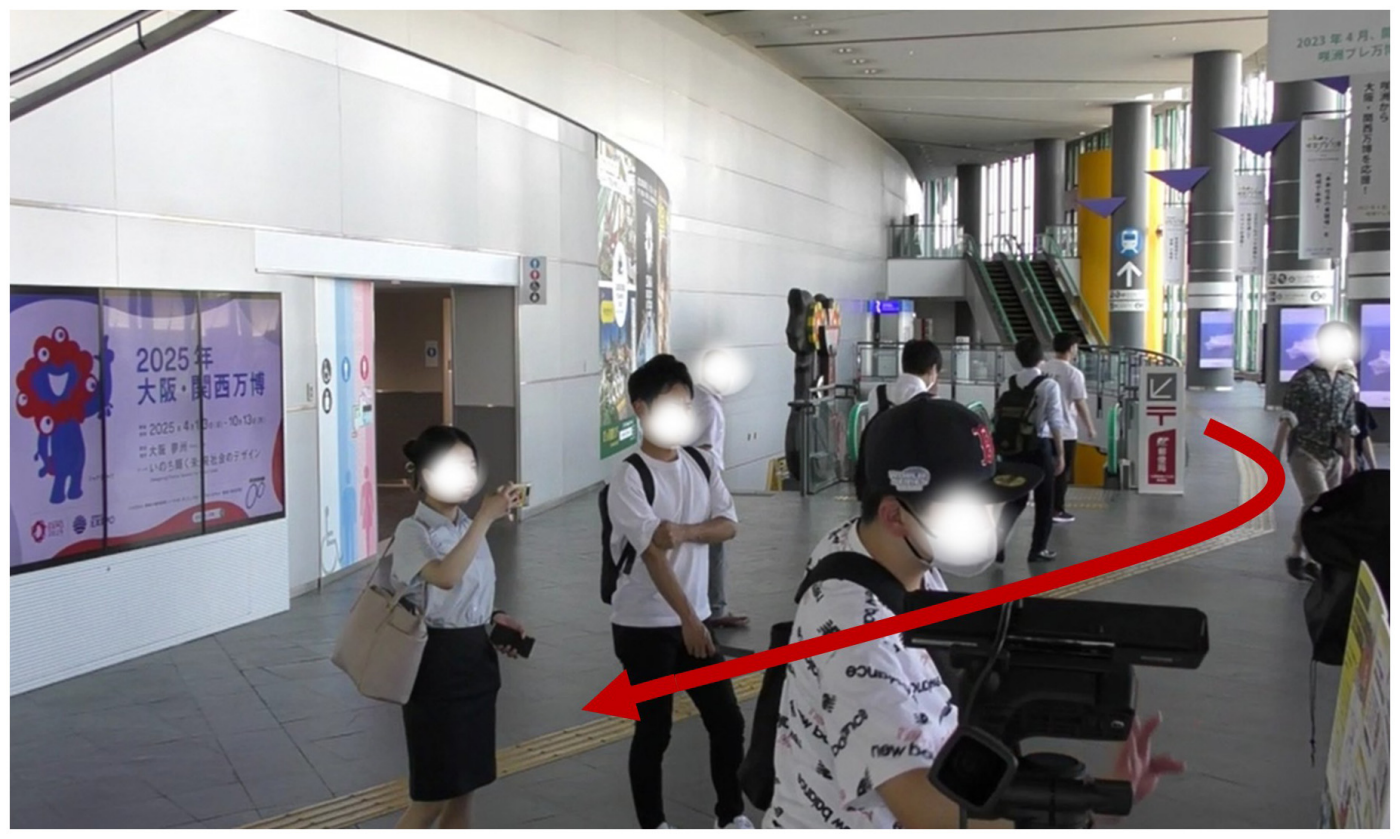
Direction of pedestrian movement included in the video annotation. Only passersby arriving from the directions indicated by the red arrows were included.

For each eligible passerby, the presence or absence of four behaviors was coded: looking at the Robot Avatar, approaching it, talking to it, and picking up a flyer. Behavioral annotations were performed using pre-defined categories for the four observable behaviors, and each behavior was coded dichotomously as present or absent for each eligible passerby. The four observable behaviors were operationally defined as follows. Looking at the Robot Avatar was coded when a passerby's face or head was oriented toward the Robot Avatar continuously for at least 1 s. Approaching the Robot Avatar was coded when a passerby moved in a direction that reduced the distance between the passerby and the Robot Avatar while their face or body was oriented toward it. Talking to the Robot Avatar was coded when a passerby was observed speaking while their face or body was oriented toward the Robot Avatar. Picking up a flyer was coded when a passerby picked up a flyer placed beside the condenser microphone and left the area with it. These measures were used as observable indicators of early-stage engagement of passersby with the Robot Avatar, including looking at it, approaching it, talking to it, and picking up a flyer.

Two independent annotators who were not involved in the research coded the video recordings. During the independent annotation, 1,315 passersby included in the final dataset were identified as eligible by both annotators, whereas an additional 64 passersby were initially identified as eligible by only one annotator. The pre-consensus records for the 1,315 passersby identified by both annotators were linked within each observation period using their chronological order and recorded arrival times, allowing a maximum difference of 5 s between the recorded arrival times. The gender and age-group categories recorded by the annotators were used as supplementary matching information. The behavioral codes were not used for record linkage.

For the 64 passersby initially identified as eligible by only one annotator, the relevant video segments were jointly reviewed and their eligibility was discussed by both annotators. Both annotators subsequently agreed to include these passersby in the final dataset. Because independent pre-consensus behavioral codes from both annotators were not available for these 64 passersby, they were not included in the behavior-specific reliability calculations. Inter-annotator reliability was therefore calculated for the 1,315 passersby for whom independent pre-consensus behavioral codes were available from both annotators. Because percent agreement can be high for low-prevalence outcomes owing to the pre-dominance of negative classifications, reliability was assessed separately for each behavioral outcome using percent agreement, Cohen's κ, and Gwet's AC1. Disagreements were defined as the number of passersby for whom the two annotators assigned different present/absent codes. Table 1 reports the cross-classification of the two annotators' codes and the behavior-specific reliability results before consensus resolution.

**Table 1:** Pre-consensus inter-annotator reliability for each behavioral outcome.

Behavioral outcome	Both present	A1 present A2 absent	A1 absent A2 present	Both absent	Disagreements (*n*)	Agreement (%)	Cohen's κ	Gwet's AC1
Looking at the Robot Avatar	683	92	76	464	168	87.22	0.737	0.751
Approaching the Robot Avatar	18	3	9	1,285	12	99.09	0.745	0.991
Talking to the Robot Avatar	5	2	1	1,307	3	99.77	0.768	0.998
Picking up a flyer	7	1	0	1,307	1	99.92	0.933	0.999

A1, Annotator 1; A2, Annotator 2. The terms “present” and “absent” indicate whether the corresponding behavioral outcome was coded as present or absent by each annotator. Disagreements are the sum of the A1 present/A2 absent and A1 absent/A2 present cells. Reliability was calculated for the 1,315 passersby for whom independent pre-consensus behavioral codes were available from both annotators.

For the 1,315 passersby independently coded by both annotators, disagreements in behavioral coding and cases that were difficult to classify were resolved through discussion and, where necessary, re-examination of the relevant video segments. The behavioral classifications of the 64 passersby initially identified as eligible by only one annotator were also determined during this consensus process. The resulting final classifications were used in the subsequent analyses. The final dataset included 479 passersby in the inter-robot interaction condition, 478 in the no inter-robot interaction condition, and 422 in the no avatar-centered social context condition (*N* = 1, 379).

## 4 Results

### 4.1 Analysis of behavioral outcomes across experimental conditions

This subsection presents the primary analyses of the four behavioral outcomes across the three experimental conditions.

The numbers of passersby who exhibited each of the four annotated behaviors were as follows: looking at the Robot Avatar, 861; approaching it, 26; talking to it, 7; and picking up a flyer, 8. Looking at the Robot Avatar was therefore the most frequently observed behavior. To examine whether the four observable behavioral outcomes differed among the three experimental conditions, we compared their occurrence rates across the inter-robot interaction, no inter-robot interaction, and no avatar-centered social context conditions. To account for multiple testing, the *p* values from the four overall tests were adjusted using Holm's method. Adjusted residuals were examined only for behavioral outcomes with a significant overall association after Holm adjustment. For these outcomes, the two-sided *p* values corresponding to the adjusted residuals across the three experimental conditions were also adjusted using Holm's method. The 95% confidence intervals for the percentage-point differences were not adjusted for multiple comparisons and are reported only to describe the magnitude and uncertainty of the estimated differences. Inferential conclusions regarding the four behavioral outcomes were based on the Holm-adjusted p-values from the four overall tests; accordingly, an unadjusted confidence interval excluding zero was not interpreted as evidence of a statistically significant condition-related difference after correction for multiple testing.

Pearson's chi-square test showed no significant association between experimental condition and looking at the Robot Avatar, χ^2^(2) = 6.643, Holm-adjusted *p* = 0.072, Cramér's *V* = 0.069 (Figure 7). The proportion of passersby who looked at the Robot Avatar was 4.05 percentage points higher in the inter-robot interaction condition than in the no inter-robot interaction condition (95% CI [−2.02, 10.07]) and 8.33 percentage points higher than in the no avatar-centered social context condition (95% CI [1.99, 14.60]). It was 4.29 percentage points higher in the no inter-robot interaction condition than in the no avatar-centered social context condition (95% CI [−2.11, 10.65]). Therefore, adjusted residuals were not interpreted for this behavior.

**Figure 7:**
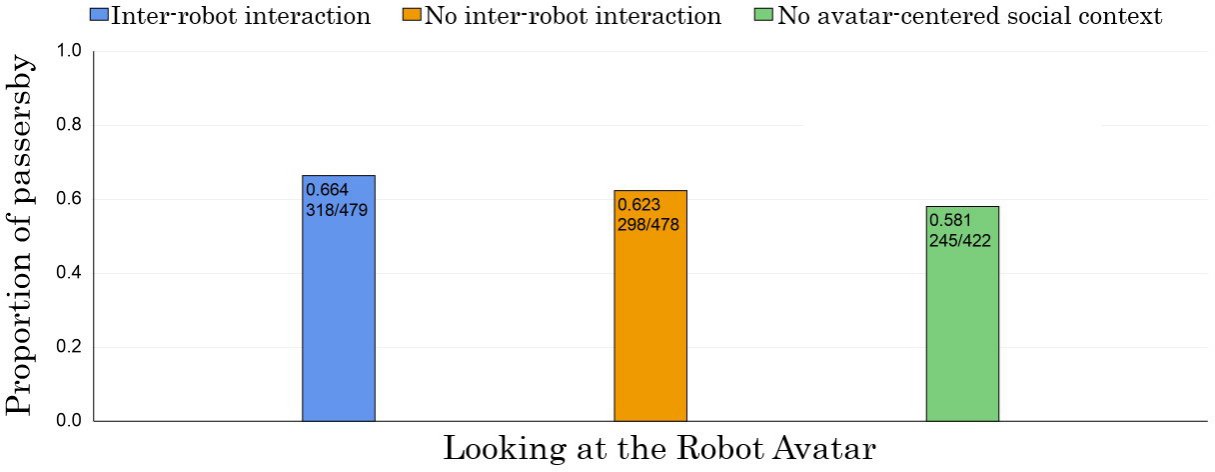
Proportion of passersby who looked at the Robot Avatar under each experimental condition.

Pearson's chi-square test showed a significant association between experimental condition and approaching the Robot Avatar, χ^2^(2) = 17.309, Holm-adjusted *p* < 0.001, Cramér's *V* = 0.112 (Figure 8). The proportion of passersby who approached the Robot Avatar was 3.34 percentage points higher in the inter-robot interaction condition than in the no inter-robot interaction condition (95% CI [1.48, 5.52]) and 3.02 percentage points higher than in the no avatar-centered social context condition (95% CI [0.98, 5.24]). It was 0.32 percentage points higher in the no avatar-centered social context condition than in the no inter-robot interaction condition (95% CI [−1.01, 1.84]). Adjusted residual analysis showed that approaching the Robot Avatar was more frequent than expected under the inter-robot interaction condition (adjusted residual = 4.15, Holm-adjusted *p* < 0.001) and less frequent than expected under the no inter-robot interaction condition (adjusted residual = −2.50, Holm-adjusted *p* = 0.025). The adjusted residual for the no avatar-centered social context condition was not significant (adjusted residual = −1.70, Holm-adjusted *p* = 0.089).

**Figure 8:**
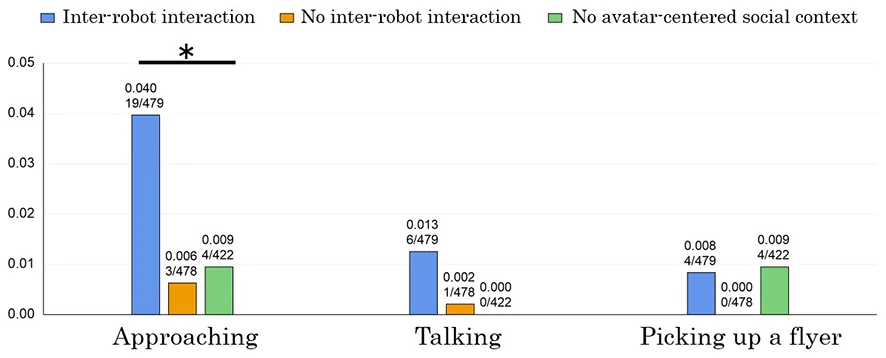
Proportions of passersby who approached the Robot Avatar, talked to it, and picked up a flyer under each experimental condition. The asterisk indicates a significant overall association (*Holm-adjusted *p* < 0.05).

Because talking to the Robot Avatar was observed only seven times, Fisher's exact test was used for this behavior. Talking to the Robot Avatar was observed in 6 of 479 passersby in the inter-robot interaction condition, 1 of 478 in the no inter-robot interaction condition, and 0 of 422 in the no avatar-centered social context condition. Fisher's exact test showed no significant association between experimental condition and talking to the Robot Avatar (Holm-adjusted *p* = 0.063). The proportion of passersby who talked to the Robot Avatar was 1.04 percentage points higher in the inter-robot interaction condition than in the no inter-robot interaction condition (95% CI [−0.14, 2.51]) and 1.25 percentage points higher than in the no avatar-centered social context condition (95% CI [0.12, 2.71]). It was 0.21 percentage points higher in the no inter-robot interaction condition than in the no avatar-centered social context condition (95% CI [−0.71, 1.18]). Therefore, adjusted residuals were not interpreted for this behavior. Given the small number of talking events, the condition-specific counts were reported descriptively.

Because picking up a flyer was also rare, Fisher's exact test was used for this behavior. Picking up a flyer was observed in 4 of 479 passersby in the inter-robot interaction condition, 0 of 478 in the no inter-robot interaction condition, and 4 of 422 in the no avatar-centered social context condition. Fisher's exact test showed no significant association between experimental condition and picking up a flyer (Holm-adjusted *p* = 0.086). The proportion of passersby who picked up a flyer was 0.84 percentage points higher in the inter-robot interaction condition than in the no inter-robot interaction condition (95% CI [−0.11, 2.13]). It was 0.11 percentage points higher in the no avatar-centered social context condition than in the inter-robot interaction condition (95% CI [−1.30, 1.66]) and 0.95 percentage points higher than in the no inter-robot interaction condition (95% CI [−0.04, 2.41]). Therefore, adjusted residuals were not interpreted for this behavior.

Table 2 presents the number of passersby and the count and percentage of each behavioral outcome for each of the nine 30-min observation periods conducted across the three experimental days.

**Table 2:** Numbers of passersby and behavioral outcomes in each 30-min observation period.

Time	Condition	Passersby *n*	Looking at the Robot Avatar	Approaching the Robot Avatar	Talking to the Robot Avatar	Picking up a flyer
A. July 12, 2023						
13:00–13:30	Inter-robot interaction	189	117 (61.9)	5 (2.6)	0 (0.0)	4 (2.1)
13:30–14:00	No inter-robot interaction	129	99 (76.7)	1 (0.8)	1 (0.8)	0 (0.0)
14:00–14:30	No avatar-centered social context	141	79 (56.0)	2 (1.4)	0 (0.0)	1 (0.7)
B. July 13, 2023						
13:00–13:30	No inter-robot interaction	225	138 (61.3)	1 (0.4)	0 (0.0)	0 (0.0)
13:30–14:00	No avatar-centered social context	120	71 (59.2)	2 (1.7)	0 (0.0)	3 (2.5)
14:00–14:30	Inter-robot interaction	190	139 (73.2)	6 (3.2)	1 (0.5)	0 (0.0)
C. July 21, 2023						
13:00–13:30	No avatar-centered social context	161	95 (59.0)	0 (0.0)	0 (0.0)	0 (0.0)
13:30–14:00	Inter-robot interaction	100	62 (62.0)	8 (8.0)	5 (5.0)	0 (0.0)
14:00–14:30	No inter-robot interaction	124	61 (49.2)	1 (0.8)	0 (0.0)	0 (0.0)

Values for behavioral outcomes are *n* (%).

### 4.2 Additional analysis accounting for clustering within observation periods

Considering that passersby observed within the same 30-min observation period may not have been statistically independent, separate binomial generalized linear mixed-effects models (GLMMs) with a logit link were fitted for each of the four behavioral outcomes. The binary occurrence (yes/no) of each behavior was set as the dependent variable, experimental condition was set as a three-level fixed effect, and the observation-period identifier was included as a random intercept with nine levels. The overall effect of experimental condition was evaluated using a likelihood-ratio test comparing the model containing condition with a model containing the same random intercept but no condition effect. When the overall condition effect was significant, pairwise comparisons were conducted; *p* values were adjusted using Holm's method, and simultaneous 95% confidence intervals were adjusted using the Bonferroni method. The GLMM results are summarized in Table 3.

Table 3Results of the binomial generalized linear mixed-effects models accounting for clustering within 30-min observation periods.

**Table:** 

Behavioral outcome	Events, *n*	Likelihood-ratio χ^2^(df)	*p*	Random-intercept variance	Intraclass correlation	Status
Looking at the Robot Avatar	861	1.664 (2)	0.435	0.066	0.020	Converged
Approaching the Robot Avatar	26	9.503 (2)	0.009	0.038	0.012	Converged
Talking to the Robot Avatar	7	—	—	—	—	Not estimable^1^
Picking up a flyer	8	—	—	—	—	Not estimable^1^
Pairwise comparisons for approaching the Robot Avatar

**Table:** 

Comparison	Odds ratio	95% confidence interval	Adjusted *p*
Inter-robot interaction vs. no inter-robot interaction	6.69	[1.41, 31.74]	0.010
Inter-robot interaction vs. no avatar-centered social context	4.41	[1.09, 17.79]	0.022
No inter-robot interaction vs. no avatar-centered social context	0.66	[0.10, 4.31]	0.596

Pairwise *p* values were adjusted using Holm's method; 95% confidence intervals were adjusted using the Bonferroni method. ^1^Not estimated reliably because a zero-event cell produced (quasi-)complete separation.

For looking at the Robot Avatar, the model converged without warnings or a singular fit, and the overall effect of experimental condition was not significant, likelihood-ratio χ^2^(2) = 1.664, *p* = 0.435 (random-intercept variance = 0.066; latent-scale intraclass correlation coefficient [ICC] = 0.020). This result was consistent with the corresponding primary analysis after correction for multiple testing.

For approaching the Robot Avatar, the model converged without warnings or a singular fit, and a significant overall effect of experimental condition was found, likelihood-ratio χ^2^(2) = 9.503, *p* = 0.009 (random-intercept variance = 0.038; latent-scale ICC = 0.012). Pairwise comparisons showed that the odds of approaching were significantly higher in the inter-robot interaction condition than in the no inter-robot interaction condition (odds ratio [OR] = 6.69, simultaneous 95% confidence interval [1.41, 31.74], adjusted *p* = 0.010) and the no avatar-centered social context condition (OR = 4.41, simultaneous 95% confidence interval [1.09, 17.79], adjusted *p* = 0.022). No significant difference was found between the no inter-robot interaction and no avatar-centered social context conditions (OR = 0.66, simultaneous 95% confidence interval [0.10, 4.31], adjusted *p* = 0.596).

For talking to the Robot Avatar, no events occurred in the no avatar-centered social context condition, and for picking up a flyer, no events occurred in the no inter-robot interaction condition. Consequently, the models for these two outcomes did not yield reliable maximum-likelihood estimates because the zero-event cells produced (quasi-)complete separation. Therefore, cluster-adjusted ORs and *p* values are not reported for talking to the Robot Avatar or picking up a flyer.

## 5 Discussion

In this study, we investigated GayaRobo as a design approach intended to enhance the social presence of a teleoperated Robot Avatar by forming a social context centered on the Robot Avatar. We examined GayaRobo in a real-world environment using four observable indicators of passersby's early-stage engagement with the Robot Avatar before persuasion or information presentation begins: looking at the Robot Avatar, approaching it, talking to it, and picking up a flyer. These behaviors differed substantially in how readily they occurred in the present field setting. Looking at the Robot Avatar was observed in more than half of the passersby under each experimental condition. The effect size for the association between experimental condition and looking at the Robot Avatar was small (Cramér's *V* = 0.069), and the association was not statistically significant after correction for multiple testing or in the additional analysis accounting for clustering within observation periods. These results do not provide clear evidence of a condition-related difference in looking at the Robot Avatar. In contrast, approaching the Robot Avatar occurred less frequently overall, but its association with experimental condition was supported by both the corresponding primary analysis and the additional cluster-adjusted analysis. This pattern suggests that, whereas looking at the Robot Avatar was common in all three conditions, approach behavior toward the Robot Avatar may have been more likely to occur under the inter-robot interaction condition. From a design perspective, approach behavior may be a meaningful observable outcome because it brings passersby into closer proximity to the Robot Avatar and may create an opportunity for subsequent interaction.

Talking to the Robot Avatar and picking up a flyer were observed only seven and eight times, respectively, and neither behavior was significantly associated with experimental condition after correction for multiple testing. Because both behaviors were rare and each had a condition in which no events were observed, the resulting (quasi-)complete separation prevented reliable cluster-adjusted estimates from being obtained. The present data therefore do not permit firm conclusions about whether the occurrence of these behaviors differed across the experimental conditions. The condition-specific counts showed that talking to the Robot Avatar was observed six times in the inter-robot interaction condition, once in the no inter-robot interaction condition, and zero times in the no avatar-centered social context condition. Although six of the seven talking events occurred under the inter-robot interaction condition, this result should be regarded as a descriptive pattern to be examined in future research rather than as conclusive evidence of a condition-related difference. In contrast, picking up a flyer was observed four times in the inter-robot interaction condition, zero times in the no inter-robot interaction condition, and four times in the no avatar-centered social context condition. Unlike talking to the Robot Avatar, picking up a flyer did not show a pattern of higher occurrence under the inter-robot interaction condition. The present data therefore do not permit a clear interpretation of the association between experimental condition and picking up a flyer. Taken together, the evidence obtained in the present study primarily concerns approach behavior toward the Robot Avatar. Whether similar condition-related patterns occur for other observable early-stage engagement behaviors, including talking to the Robot Avatar and picking up a flyer, requires further investigation. Future studies should also examine interaction designs intended to support the initiation of dialogue in addition to approach behavior. One possible direction is to investigate whether adapting the content and timing of the Gaya robots' responses based on passersby's observable engagement behaviors could support the transition from early-stage engagement with the Robot Avatar–particularly approach behavior–to the initiation of dialogue. This line of research could clarify whether and under what conditions approach behavior is followed by dialogue, thereby informing the application of GayaRobo to persuasive interaction.

The observed behavioral pattern, most clearly reflected in the association between the inter-robot interaction condition and approaching the Robot Avatar, may be interpreted from the perspective of social proof. Under this interpretation, the Gaya robots' responses can be conceptualized as presenting the Robot Avatar as the target of the surrounding robots' attention and responses, although the present study did not assess whether passersby perceived the interaction in this way. This account draws on the concept of social proof, which suggests that people tend to direct their attention toward objects attended to by others (Cialdini, 2013), and on previous research indicating that passersby tend to direct their attention toward objects attended to by robots (Amada et al., 2023). However, the present study did not quantitatively measure or match the amount and frequency of speech and movement across conditions. With the intention of reducing differences in the amount of speech across conditions, the Gaya robots delivered a succession of pre-prepared utterances throughout each observation period in the no avatar-centered social context condition, with no intervals during which both robots were silent. Nevertheless, aggregate speaking time and the total number of utterances across all three robots were not quantified in either the inter-robot interaction condition or the no avatar-centered social context condition; thus, the difference in overall speech activity between these conditions remains unclear. The perceptual salience of speech and movement was also not measured. The present experimental design therefore cannot determine whether, or to what extent, the observed association reflects a process consistent with social proof, the effects of uncontrolled differences in robot activity across conditions—including the amount, frequency, novelty, and perceptual salience of speech and movement—or both. Accordingly, social proof should be regarded as one possible theoretical account of the observed association rather than as an established mechanism. The present findings nevertheless motivate future research to test whether an avatar-centered social-context design can influence observable early-stage engagement behaviors through a process consistent with social proof.

The present study broadens the design focus beyond the appearance and utterance content of the Robot Avatar itself to include the social context surrounding it as an additional design consideration. In previous studies on Robot Avatars and social robots, methods for improving users' evaluations of robots as social interaction partners, such as social presence and trust, have been investigated by designing and manipulating social cues presented by the robots themselves, including appearance, voice and language, bodily movements, facial expressions, and gestures (Bremner and Leonards, 2016; Tanaka et al., 2015; Xu et al., 2023). In contrast, this study adopted a robot-to-robot interaction approach in which the Gaya robots responded to the Robot Avatar; this approach was intended to present the Robot Avatar as a social entity situated within relationships with the surrounding robots. In addition, Gaya robots do not function as independent agents that directly approach passersby, but rather as supporting entities for the Robot Avatar. This design concept may be relevant to the development and evaluation of persuasive interaction systems at the initial stage before information presentation or persuasion begins.

The contribution of this study lies in proposing GayaRobo as a design approach intended to enhance the social presence of a teleoperated Robot Avatar by forming an avatar-centered social context, and in empirically examining the association between the inter-robot interaction condition and observable early-stage engagement behaviors in a real-world customer service setting. The findings most clearly support an association between the inter-robot interaction condition, as implemented in this study, and approaching the Robot Avatar. On this basis, approach behavior may be a relevant observable outcome for future evaluations of Robot Avatar systems incorporating avatar-centered social-context designs, although the specific contribution of the social-context component remains to be established. More broadly, the study informs future research on persuasive interaction design in customer service settings, including advertising, guidance, recommendation, and customer interaction. Its field setting in a commercial facility also provides practical insights into evaluating such designs under real-world operating conditions.

## 6 Limitations

This study has several limitations. First, the additional GLMM analysis was based on only nine 30-min observation periods, with three observation periods per condition across three experimental days. This small number of clusters limits the precision of the estimated condition effects and random-intercept variances and may make the associated p-values and confidence intervals unstable. A GLMM including random intercepts for both experimental day and observation period was also examined. However, the estimated day-level random-intercept variance was zero or close to zero for all four behavioral outcomes, resulting in singular fits. Thus, the present data did not provide sufficient information to estimate day-level variation separately from observation-period-level variation. The results of the additional analysis should therefore be interpreted cautiously. In addition, talking to the Robot Avatar and picking up a flyer were observed only rarely, and each of these outcomes had a condition in which no events were observed. The resulting (quasi-)complete separation prevented reliable cluster-adjusted estimates from being obtained for these two behaviors. Future studies should include more experimental days and observation periods and collect sufficient behavioral events to permit more reliable cluster-adjusted estimation.

Second, although the order of the three conditions was rotated across the three experimental days so that each condition was implemented once in each of the three time slots, transitions between conditions were not fully counterbalanced. Therefore, possible carryover from the preceding 30-min observation period cannot be excluded. In addition, this was not a repeated-measures design; passersby were sampled during the observation periods assigned to each condition rather than intentionally followed or matched across conditions. Consequently, unmeasured differences in passerby characteristics across observation periods, such as whether passersby were walking alone or in groups, their reasons for visiting the facility, or their prior interest in robots, may have influenced the results. Although the additional GLMM with a random intercept for observation period accounted for the non-independence of observations within the same observation period, it did not separately adjust for possible carryover from the preceding observation period or these unmeasured differences in passerby characteristics. Therefore, the findings regarding the associations between experimental condition and the behavioral outcomes should be interpreted cautiously because these observation-period-level influences could not be fully separated from the experimental condition.

Third, we did not directly measure social presence. Instead, we assessed passersby's observable early-stage engagement using four behavioral indicators: looking at the Robot Avatar, approaching it, talking to it, and picking up a flyer. Therefore, the present findings alone do not establish that the proposed approach enhanced the social presence of the Robot Avatar. Previous research has reported an association between questionnaire-based measures of social presence and user engagement (Jin et al., 2017), indicating the value of examining the relationship between social presence and engagement. However, because this association was observed between self-reported measures, the relationship between the observable early-stage engagement behaviors examined in the present study and passersby's perceived social presence remains unclear. A related limitation concerns the interpretation of looking at the Robot Avatar as an indicator of early-stage engagement. Although looking at the Robot Avatar may be consistent with early-stage engagement, it may also reflect general visual attention or novelty-related responses. In the present study, this behavior occurred in more than half of the passersby under all three conditions, showed only a small association with experimental condition (Cramér's *V* = 0.069), and was not significantly associated with condition after correction for multiple testing or in the additional analysis accounting for clustering within observation periods. Therefore, the present data do not allow us to determine whether, or to what extent, the observed looking behavior reflected early-stage engagement rather than general visual attention or novelty-related responses. Looking behavior may thus have had limited specificity as an indicator of early-stage engagement associated with the proposed interaction design in this field setting. Future studies should therefore combine behavioral observations with direct assessments of passersby's perceived social presence of the Robot Avatar using questionnaires or interviews, and examine both whether the proposed approach affects perceived social presence and whether perceived social presence is related to observable early-stage engagement behaviors.

Fourth, the inter-robot interaction condition differed from the comparison conditions not only in the temporal contingency of the Gaya robots' responses to the Robot Avatar's utterances but also in the amount and frequency of speech and movement produced by the Gaya robots. The present experimental design therefore does not allow the observed difference in approaching the Robot Avatar to be attributed specifically to the Gaya robots' contingent responses rather than to differences in the amount, frequency, novelty, or auditory and visual salience of their speech and movements. Accordingly, this finding should be interpreted as an association with the inter-robot interaction condition as implemented as a whole, and the specific contribution of the avatar-centered social-context design remains uncertain. Future studies should include a matched noncontingent condition in which the same utterances and movements by the Gaya robots are presented at comparable frequencies but are not temporally linked to the Robot Avatar's utterances. Such a condition would help distinguish the contribution of contingent robot-to-robot interaction from the possible effects of differences in auditory and visual activity. More broadly, future studies should separately examine other design factors, including the timing of the Gaya robots' responses, the salience of their speech and movements, and the number of robots, to clarify their relative contributions to observable early-stage engagement behaviors.

Fifth, the present study did not examine whether the specific puppet-like appearances of the three robots influenced the observed behavioral patterns. Although each robot's appearance was held constant across all three conditions, the sheep and dog appearances of the Gaya robots and the black clothing and sunglasses of the Robot Avatar may have influenced passersby's overall responses; in particular, the sunglasses may have been visually salient in an indoor commercial setting. Previous research has shown that a robot's appearance can affect social judgments such as likability and trust (Mathur and Reichling, 2016). Accordingly, the generalizability of the present findings to robots with different appearances remains unclear. Future research should examine whether a similar association between experimental condition and approaching the Robot Avatar is observed when robots with different appearances are used.

Sixth, because the Gaya robots in this study used only pre-recorded brief utterances, users' engagement may decrease during long-term operation due to repeated responses or contextual mismatches. Future studies should examine interaction designs that use large language models to generate context-appropriate responses while maintaining the Gaya robots' supporting role for the Robot Avatar. Such designs may allow the Gaya robots to adapt their responses more flexibly to the ongoing situation while preserving an avatar-centered social context.

## 7 Conclusions

In this study, we proposed GayaRobo as a design approach intended to enhance the social presence of a teleoperated Robot Avatar by forming an avatar-centered social context through robot-to-robot interaction. In GayaRobo, two Gaya robots are placed around the Robot Avatar and respond to the Robot Avatar via speech and movement, rather than approaching passersby as independent agents. To evaluate this approach, we conducted a field experiment in a commercial facility and examined observable behavioral indicators of passersby's early-stage engagement with the Robot Avatar. Both the primary analysis and the additional cluster-adjusted analysis indicated a significant association between experimental condition and approaching the Robot Avatar. Adjusted residual analysis indicated that approaching the Robot Avatar was more frequent than expected under the inter-robot interaction condition and less frequent than expected under the no inter-robot interaction condition.

This finding pertains to the inter-robot interaction condition as implemented as a whole because the present experimental design neither quantitatively matched overall robot activity, including speech and movement, across conditions nor isolated the specific contribution of the avatar-centered social-context component. Because social presence was not directly measured, the behavioral outcomes should be interpreted as indicators of early-stage engagement rather than as evidence that GayaRobo enhanced the Robot Avatar's social presence. Taken together, these findings highlight approach behavior as a relevant observable outcome for future controlled evaluations of Robot Avatar systems incorporating avatar-centered social-context designs. Future research should isolate the specific contribution of the avatar-centered social-context component from differences in overall robot activity and directly examine how this component relates to both approach behavior and passersby's perceived social presence of the Robot Avatar.

## Data Availability

The raw video data are not publicly available due to privacy and ethical restrictions, while the anonymized behavioral annotation data and aggregated data are available from the corresponding author on reasonable request.
